# Assuring patient participation and care continuity in intermediate care: Getting the most out of family meetings using the four habits model

**DOI:** 10.1111/hex.13591

**Published:** 2022-08-23

**Authors:** Linda A. H. Kvæl, Cecilie F. Olsen

**Affiliations:** ^1^ Department of Rehabilitation Science and Health Technology, Faculty of Health Sciences Oslo Metropolitan University Oslo Norway; ^2^ Department of Ageing Research and Housing Studies, Norwegian Social Research—NOVA Oslo Metropolitan University Oslo Norway

**Keywords:** care continuity, clinical tool, communications skills, family meetings, four habits model, intermediate care, patient participation

## Abstract

**Introduction:**

As a transitional care intervention, intermediate care (IC) bridges the pathway for older patients from hospital to home. Within family meetings in IC, the older patient, his or her relatives, the interdisciplinary team and the municipal case manager come together to discuss goals and interventions during the IC stay, including follow‐up services after discharge. Although family meetings are a common aspect of teamwork in IC, it is unclear how the voices of older people and their relatives are coming across. The aim of this study is to explore how patient participation is framed and negotiated within family meetings in IC.

**Methods:**

This study is based on qualitative data from the observation of 14 interdisciplinary family meetings in Norwegian IC services. As a theoretical framework, the authors have used the four habits model developed by Frankel and Stein as a lens to understand the interrelated sequence of events that typically takes place during a family meeting and the importance of communication skills to promote patient participation.

**Results:**

The thematic analysis resulted in 16 categories and 4 main themes related to the 4 habits model: (i) grounding the family meetings, (ii) what matters to you?, (iii) being empathically present and (iv) the power of a final closure.

**Conclusion:**

There was considerable variation in the way current family meetings were conducted. It seemed crucial to start the meeting with a proper introduction and explanation of the purpose of the meeting to establish trust and to be able to successfully move to the next stage of eliciting the patients' preferences, views and goals via the ‘what matters to you?’ question. There were examples of empathetic communication among meeting participants perceived to facilitate patient participation. Finally, to successfully end the meeting and agree on a shared plan, it seemed crucial for case managers who held the decision‐making power to attend the meetings. Framing family meetings in line with the four habits sequential approach may have the potential to assure patient participation and care continuity in IC services.

**Patient or Public Contribution:**

This article is part of a larger project based on a James Lind Alliance process that brings patients, relatives, health care professionals and researchers together in priority setting partnerships to identify and prioritize evidence uncertainties that they agree are the most important. Accordingly, the design and content of this article have been initiated and discussed in the project's stakeholder group consisting of one patient representative, one relative representative, two health care professionals from IC settings, two representatives from the Norwegian Health Association and two representatives from the Agency of Health in Norway.

## 1 INTRODUCTION

As a transitional care intervention, intermediate care (IC) aims to provide continuity of care for older patients from hospital to home. It entails rehabilitation for a short time period, either after hospitalization or to prevent hospital admissions, within or nearer to people's homes.^1^ Due to early hospital discharge, these services are highly valued^2^ and important in enabling older persons to live in their own homes for as long as possible.^1^

Successful family meetings are a cornerstone to obtaining patient participation in health care.^3^ Within family meetings in IC services, the older patients and their relatives come together with the interdisciplinary team consisting of health care professionals (HCPs) and the municipal case managers (CMs) to discuss goals and interventions during the IC stay, including length of stay and follow‐up services after discharge, to provide continuity of care.^4^ This is expected to contribute to different dimensions of continuity of care: *relational continuity* entailing a trusting relationship with HCPs over time, *informational continuity* entailing timely sharing of patient‐related information and *management continuity* entailing the communication about patient‐relevant information across teams, HCPs and institutional barriers.^5^, ^6^, ^7^ However, although family meetings are a common aspect of teamwork in IC, how the voices of older people and their relatives are negotiated is unclear.^8^, ^9^, ^10^ True patient participation is a complex process that needs to be framed competently,^11^ and a greater exploration of how patient participation can be negotiated is called for.^4^, ^9^, ^10^

Patients are entitled to participate in the design and delivery of their own health care.^9^, ^12^, ^13^, ^14^ Patient participation in the context of IC can be defined as—a dynamic process emphasizing the person as a whole, focusing on the establishment of multiple alliances that facilitate individualized information and knowledge exchange, and ensuring a reciprocal engagement in activities within flexible and interactive/dynamic organizational structures.^13^ Asking the question ‘What matters to you?’ has become a popular method in the implementation of patient participation, embracing recognition and seeing the person behind the diagnosis. However, using the question is challenging and requires relational competence.^11^ Research has shown that a professional's attitude and tone of voice determine the patient's involvement,^15^ and important aspects of person‐centred communication include actively listening to a patient's narrative as well as being sensitive to their cues and concerns.^16^ Patient participation is nested within a network of complex relationships. Accordingly, teamwork must be built on shared knowledge and goals, mutual respect^17^ and trust to hand over responsibility to others.^18^ According to van Dongen et al.,^19^ in successful family meetings, the mutual relationships between HCPs, patients and relatives should be based on trust and equality to provide a comfortable atmosphere and relational continuity. Hence, HCPs' approachable attitudes when framing the meetings seem to be of relevance.^19^

### 1.1 The four habits model

The four habits model (FHM) by Frankel and Stein^20^ consists of four interrelated domains of how to structure a clinical encounter, where the different communication tasks that embrace each habit are organized into groups of skills, techniques and pay‐offs. The first habit is to invest in the beginning. Here, three tasks must be accomplished to be successful: quickly build trust, explore the older person's concerns and plan the stay or meeting. The second habit is to elicit the patient's perspective (e.g., using the question ‘what matters to you?’). The third habit includes demonstrating empathy and being aware of the patient's cues and nonverbal signals. Finally, the last habit is to invest in the end by providing information and encouraging patients to participate in decision‐making while negotiating treatment and follow‐up plans.^20^ The FHM is well documented and validated for use in general practice and hospital doctor encounters^21^, ^22^ among patients in emotional distress,^23^ but it has not been explored in family meetings in an IC context. The model appears to be a simple and teachable scheme for structuring family meetings to promote successful patient participation.

### 1.2 Aim of the study

Although patient participation has been recognized as a strategy to achieve person‐centred care,^24^ implementation of patient participation in everyday practice is hard to achieve and requires professional competence and practice.^11^ Fortunately, the literature highlights that clinical communication skills can be thought, learned and practiced.^20^ However, many HCPs receive little or no training in the context of IC. If used efficiently, a family meeting has the potential to be a powerful tool for promoting patient participation in care.^4^ In a recent Cochrane review, the authors concluded that decision‐coaching (i.e., when HCPs help patients to actively participate in making decisions) may improve the patient's knowledge, but further research is needed.^25^ Thus, the aim of this study is to explore how patient participation is framed and negotiated within family meetings in IC services using the FHM. This may inform future strategies for improving and implementing patient participation in this setting.

## 2 METHODS

This article is based on qualitative data from the observation of 14 interdisciplinary family meetings in 3 bed‐based IC institutions in Norway. In a previous article, the use of different positions to get one's point across in the meetings was reported.^4^ In this article, we use the FHM to explore how patient participation is negotiated and framed within family meetings in IC. The study is theory‐driven and informed by a social constructivist approach.

### 2.1 Context of the study

In Norway, health care is organized into specialized and primary care levels.^26^ Accordingly, Norway has established IC services in recent decades to ensure continuity of care. As a new public management strategy and to promote efficiency, a purchaser–provider model has been established in which the purchasers have the administrative authority to approve the services, while the providers have little influence other than performing the services.^27^, ^28^ According to the IC routine, a family meeting should be held 2–3 days after patient admission to the IC and should last no more than 30 min. The main objective of these meetings is to obtain an accurate and safe start‐up for the patient. In addition, the intention is to create a rehabilitation plan and discuss the length of stay as well as follow‐up services after discharge to promote continuity. According to the routine, the patient, their relatives, the IC team and the municipal CM should be present. The patient's primary nurse is responsible for organizing, inviting and leading the meetings, obtaining necessary information in advance and documenting and coordinating further progress.

### 2.2 Characteristics of family meetings

Of the 14 observed family meetings, eight took place in patients' rooms, five in the open kitchen/living room area and one meeting in a conference room (see Table 1). The 14 patients included 5 men and 9 women; they had an average age of 87 years, and 4 had obtained higher education. Patients' relatives, aged between 50 and 87 years, participated in 9 of the meetings. The relatives included seven daughters, four sons, a sister and a daughter‐in‐law. Most commonly, the IC team present comprised a nurse and/or an auxiliary nurse, an occupational therapist and/or a physichal therapist. There was no organized training to lead a family meeting in IC. A checklist had been developed, but this was not used in any of the observed meetings in this study. The municipal CMs were present in nine of the meetings. Overall, the meetings were held on the 5th or 6th day after the transition to IC, and they lasted between 10 and 56 min, with an average of 32 min.

**Table 1:** Characteristics of family meetings

FM	Characteristics of the patients	Relatives	IC team	CM	Time of duration	Place of the meeting	Time of completion	District
1.	Female, 90 years, E = High	Two daughters	Nurse	x	30 min	In the patient room	After 1 week	1
2.	Male, 85 years, E = Low	Sister	Nurse, nursing assistant		40 min	In the patient room	After 3 days	2
3.	Female, 92 years, E = Low		Nursing assistant, PT and OT	x	47 min	In a conference room	After 2 weeks	1
4.	Female, 97 years, E = Low	Daughter‐in‐law and son	Nurse, PT and OT	x	33 min	In the patient room	After 3 days	3
5.	Female, 85 years, E = Low	Daughter	Nurse, PT and OT	x	27 min	In the patient room	After 2 days	3
6.	Male, 83 years, E = Low	Daughter	Nurse, PT and OT		56 min	In the patient room	After 6 days	4
7.	Male, 76 years, E = Low		Nurse, OT and PT student	x	10 min	In the patient room	After 2 days	5
8.	Female, 94 years, E = Low		Nursing assistant, PT and OT		20 min	In the patient room	After 2 days	5
9.	Female, 74 years, E = Low		Nurse, PT, OT and student		32 min	In the patient room	After 2 days	6
10.	Male, 89 years, E = High		PT and OT (two from the district)	x	28 min	At the kitchen/living room	After 6 days	7
11.	Female, 88 years, E = Low	Daughter	Nursing assistant and PT	x	40 min	At the kitchen/living room	After 6 days	8
12.	Male, 88 years, E = High	Daughter	Nurse, PT, OT and student		30 min	At the kitchen/living room	After 4 days	7
13.	Female, 86 years, E = High	Daughter and son	Nurse and PT	x	40 min	At the kitchen/living room	After 1 week	7
14.	Female, 91 years, E = Low	Two sons	OT and two from the district	x	20 min	At the kitchen/living room	After 2 weeks	7

Abbreviations: CM, case manager; E, education; FM, family meeting; IC team, the interdisciplinary team; OT, occupational therapist; PT, physical therapist.

Lower education: Elementary school and high school. Higher education: University and college.

### 2.3 Data collection

The observational data consisted of meeting notes and audiotapes. Initially, we recruited older persons through strategic sampling to reflect the heterogeneity in IC services. We included older persons over 65 years who were transferred to IC from hospital or home; they were dependent in activities of daily living but had an overall aim of being able to stay at home. HCPs in IC provided information about the project. The older persons willing to be included were given more nuanced information. Then, with the patients' consent, the informal caregivers were invited to take part by the first author. Finally, HCPs and the municipal CM were contacted and invited to participate. The first author observed the family meetings without participating and took notes. All meetings, held between April and December 2017, were recorded and transcribed verbatim by the same researcher. Both researchers have backgrounds as physical therapists (PTs) and have broad clinical experience with IC services as well as research experience with transitional care pathways for older people.

### 2.4 Data analysis

To analyse the transcribed data material and meeting notes, we used thematic analysis in line with Braun and Clarke.^29^ Initially, the first author read the transcribed data as open as possible, exploring meanings and insights and, in accordance with the notes, summarizing the emerging patterns of what happened during the meetings. The data were then coded by the first author and grouped into initial themes. At this stage, it became apparent that the meeting structure had a great influence on the success of patient participation. Accordingly, we realized that the FHM could serve as a suitable lens when abstracting data more deductively into further analysis, resulting in four main themes representing the four habits (Table 2). The themes were discussed elaborately among the authors to ensure that they were underpinned by quotes from the data material. Finally, to enhance validity, the first author discussed the findings with older persons and informal caregivers, as well as with a stakeholder group.

**Table 2:** The analysis

Categories of codes	Main themes
Establishment of alliances	Grounding the family meetings
Individualized information	
Clarification of expectations	
Group composition	
The patient narrative	What matters to you?
Translation of goals	
Exploring patient preferences	
Contextual concerns	
Being sensitive to patients' cues	Being empathically present
Non‐verbal expressions	
Understanding emotions	
Tense words and metaphors	
Summary and conclusion	The power of a final closure
Explore patient comprehension	
Shared decision‐making	
Clarification of responsibilities	

### 2.5 Ethical considerations

The study was pre‐approved by the Norwegian Centre for Research Data (No. 53013). All informants gave their consent to participate after they had received individualized and sufficient information. This also included the possibility of taking back the consent to participate. All recorded data material was stored in Services for Sensitive Data, as suggested by Norway's Privacy and Electronic Communication Directive. In the following results, all informants are pseudonymized to preserve the principle of anonymity.

## 3 RESULTS

The thematic analysis (Table 2) resulted in 16 categories of codes and 4 main themes: (i) grounding the family meetings, (ii) what matters to you?, (iii) being empathically present and (iv) the power of a final closure.

### 3.1 Grounding family meetings

Overall, there was no consensus on how to start family meetings. In some cases, the patients and their relatives were prepared in terms of the purpose and function of the meeting. In other cases, however, the interdisciplinary team went straight to the point, asking the patient ‘what matters to you?’, leaving the patient in an undoubtedly frustrating situation with no clue what to answer. A clear observation was that an investment at the beginning of the meeting lay the premises for patient and relative participation. One of the CMs seemed to achieve trust and respect by addressing the patient as Mrs Jones and further by making a nonmedical comment to put the patient at ease, such as ‘How beautiful flowers you have Mrs Jones? Lovely autumn flowers are they Asters?’. Furthermore, after some small talk and introducing everyone in the room to create rapport, the results suggest the importance of eliciting the patient's (P) concerns by asking open‐ended questions. In one of the meetings, the PT had an open scene like this:

PT: Yes, welcome to this family meeting. We tend to gather the troops, simply to agree on what next, how it has been before the current [hip fracture] and…yes, status today. Decide simply. Would like to hear from all parties really what they think and how we should do it further [turning to the patient]. But, Mary, how do you think it is going?

P: Really good, I have even tried the stairs… So, I think it is going in the right direction [silence]. But I need help in the morning care…

PT: Hmm…go on…[silence].

P: To get in and out of bed and to put on trouser, cause my leg won't listen to me…

In the above example, the PT allows silence to let the patient think and uses an encouraging expression, such as ‘Hmm, go on…’, inviting the patient to explain more in depth. In another meeting, a similar introduction was used, but here, the nurse did not set the agenda for the meeting (as clearly). This meeting lasted for approximately 1 h, where the daughter did most of the talking, mostly about herself, and where no chair of the meeting intervened. Hence, overall, it seems that matching of expectations may help plan family meeting(s). Furthermore, the attendees of the meeting seem to be crucial. Also, in addition to the patient, the IC team, CM and relatives should be present to promote the patient's vulnerable voice. However, this was not the case in one‐third of the meetings.

### 3.2 What matters to you?

Despite widely different initial approaches, most family meetings involved asking the question ‘what matters to you?’ for determining the patient's goals in the rehabilitation process. In one of the family meetings, the nurse (N) and the CM elicited the patient's perspective like this:

N: So, we wonder what matters to you in everyday life at home?

P: Currently, it's getting back on my feet and being able to help at home. To help my wife, she has trouble walking, and…she also has dementia, not able to do anything by herself anymore. You know…she forgets the kettle, and…

N: Yes, when making food, I understand [empathic facial expression]. Okay, but what do you think you have to practice here to return home to your wife?

P: To be able to walk independently again.

OT: Okay, so how do you live, Henry?

P: I live in an apartment with three rooms, third floor.

OT: With an elevator?

P: No…(laugh), no elevator, unfortunately.

CM: And I guess there are some thresholds, right?

P: Yes, especially between the hallway and the bathroom. That's where I broke my leg.

OT: I see, do you usually use any aids when walking inside?

P: Yes, I use a walker.

OT: And outside?

P: I haven't been outside for a long time. I am not able to do the stairs anymore. Regarding groceries, we get help from our son and daughter.

CM: […] Okay, I think we will do a home visit together with you [looking at the patient]. When seeing your home, we can plan and see if there is anything we can do to make your home safer, enabling you to move around and to leave the apartment.

The above conversation illustrates the relevance of exploring the patient's point of view, both in terms of what they believe causes their situation and their preferences about what is likely to help. The patient in the above example has a clear physical goal (i.e., being able to live home with his wife), which means that he must be able to walk independently. Hence, HCPs might translate this goal into subgoals with adjoining measures such as leg strength training, balance training and stair training. In addition, they will equip the patient's home environment to prevent further falls. Consequently, as illustrated in several of the meetings, the exploration of the larger context surrounding the patient's life seems to be crucial to understand the impact of the current medical issue or more immediate reason for being in IC. An important observation was that eliciting patients' perspectives provided new insights into the problem, as it has the potential to uncover hidden concerns. However, the success of exploring a patient's views depends clearly on the initial grounding of the meeting.

### 3.3 Being empathically present

A crucial aspect, lacking in several of the family meetings, was being sensitive to the patients' cues, understood here as the nonverbal language or cues spoken in tense words or metaphors. Accordingly, being empathically present seems to be a necessary skill to succeed. In several of the meetings, patients or relatives provided the situation with cues of hidden emotions not captured by the IC team. For example, in one of the meetings, an older male patient stated:

P: So, I believe that's status quo!

PT: Okay, fine, do you have any further questions, Phillip?

P: No… [hesitates a bit], otherwise it's a very modern place and room and…but…

CM: Yes, this place is newly renovated, in February, so that is not a long time ago […]

P: However, I have the impression that this institution here is like many others; it is organized primary to make money… I don't know if that's just my opinion…? But you might think about that…

All: [Insecure laughter followed by an awkward silence].

OT: Well, it does not benefit my pockets at least, so I don't know…[laughter].

PT: Okay, so if there are no more questions, I believe we can… Lunch…

P: Well, I believe there were a few questions in my last comment…

CM: Yes, now we have an overview of your situation, and we will take it from here…

As illustrated above, the HCPs either do not capture the patient's ‘invitation’ to discuss the quality of care he receives, or even worse, they ignore it. After the family meeting, the patient revealed, among other things, poorly cooked food served in an unworthy manner. These are issues that could have been addressed and easily changed. Our results indicate that HCPs need to practice openness towards patients' emotions, verbally and non‐verbally, and be aware of their own reactions when, for example, confronted with unpleasant comments. In contrast, during another meeting, the CM is consistently observant of how the patient is feeling, speaks loudly and calmly, addresses the patient directly and repeats what the other teammates say over the patient's head. In this way, the CM embraces the patient's vulnerability in an empowering and sensitive way, demonstrating empathy both verbally and nonverbally:

OT: I can say, I've ordered a stove guard and a toilet raiser with armrests [informing the CM with a rather low voice].

CM: Mmm… [looking at the patient and raising her voice], we are talking about the aids to your home to make it safer.

P: Yes, I've been talking about that earlier with him [pointing to the PT].

CM: Yes, and now they are telling me what you will need when you get home.

P: I find the toilet good as it is, actually. But they want me to use a toilet raiser.

CM: Mmm, it is often very wise because it gets easier to stand up as well as to sit down, so it might prevent any further fall accidents.

P: Yes, that's true, that is true!

CM: Because we want to help you to stay at home for as long as possible.

P: Oh, yes, I hope so.

CM: Yes, I understand that is what matters to you […].

P: I had hoped to be able to go home today because I panic at night…

CM: Yes [empathic facial expression]. But you know, Friday afternoon is never a good day to go home… It is better to wait until next week…

P: I don't understand how to handle it, don't understand [frustrated voice].

CM: But, Jenny, what can the HCPs here do so that you do not feel that panic at night?

P: I don't know; I just want to go home.

CM: Do you use the dial when you panic?

P: Yes, and the people working here are very nice.

CM: Are they able to calm you down?

P: Yes, they help me to the toilet and over in the chair, and I sit there for a while, then they help me back into the bed again.

CM: You see, we really need to plan and prepare your homecoming. Do you understand why we want to keep you here until Monday?

P: Yes, you are concerned about my safety.

CM: [looking at the IC team while speaking loudly to inform the patient] Please have a conversation together with Jenny, and maybe include the doctor, and explore together how you can make the two last nights more comfortable.

### 3.4 The power of a final closure

The power of a final closure implies summing up the meeting while checking the patient's comprehension. It seemed that clarification of responsibilities through shared decision‐making may provide a clear understanding and agreement on courses of action after the IC discharge. Family meetings without the CM (i.e., the one with administrative authority) had commonly nothing to conclude, since no one present could make any decisions about follow‐up services. For example, in one family meeting, the patient and his daughter (D) were terrified about being prematurely discharged home. Although the HCPs invested in the beginning and elicited the patient's perspective while demonstrating empathy, they were not able to close the meeting sufficiently due to the lack of administrative authority, which rather created loose threads, unanswered questions and concerns about the future.

OT: Okay, I believe we must end the meeting now. We will conduct a home visit with you [looking at the patient] next week. We will then let the CM know how it turns out, and they [the municipal] will extend your stay if they find it necessary.

P: We would really like to promote such an extension of my stay here…

D: And I guess you [looking at the IC team] will support that?

OT: We can only describe his situation, both physically as well as the insecurity, and his home environment. We are not allowed to suggest anything… We can only describe…

D: Okay, it's the municipal case manager that has the administrative authority, so it's they we must call and…argue…?

OT: Yes, they decide, and as I usually say: you must take it with them.

P: However, they are not here, and they are not easy to reach… [frustrated tone].

Consequently, our findings clearly suggest that, in addition to the patient, the relatives, the IC team and the CM should be present to enable real decision‐making activities. This was the case in 9 of the 14 meetings that we observed. Contrastingly, in another meeting, the patient, the relatives, the IC team and the CM were present. Here, after a nice presentation of all the participants and after exploring the patients' and relatives' points of view in an empathic way, the CM provided in‐depth and comprehensible information about physical and social follow‐up services. Building on the previous themes, the CM was thus able to close the meeting in a good way.

OT: Okay, to sum up, we will make a home visit with you and your son, and we will provide you with the necessary aids enabling you to go home next week as planned.

P: That sounds like a good plan. I really appreciate that.

CM: A team will follow you up after discharge to establish continuity in training. When you feel better, it's a good idea to return to the day centre, and a car from the volunteer centre can pick you up if you like. Do you have any further questions?

P: No, I believe that everything is clear now, thank you for this informative meeting.

All the family meetings observed contained, to varying degrees, dimensions from the included themes; however, only a few framed them all. In that way, the four themes seem to be interrelated, meaning that they build on each other as stepping stones to structure the family meetings in a competent and successful way.

## 4 DISCUSSION

This study aimed to explore how patient participation was framed and negotiated within family meetings in IC services using the FHM.^20^ We observed considerable variation in how the family meetings in IC were organized and conducted in terms of all four habits. In this section, we will discuss how the results related to the FHM^20^ and what possible practical implications a clearer structuring of IC family meetings in line with the model may have for patient participation and care continuity in this setting.

Overall, our findings are in line with prior research exploring FHM in other health care settings,^21^, ^22^, ^23^, ^30^, ^31^ and the elements of the model seemed to point out crucial clinical communication skills to promote patient participation as well as continuity of care in IC.

The first theme, ‘grounding the family meetings’, is closely related to the first habit of ‘investing in the beginning’, which, in the FHM, consists of quickly creating a rapport with the patient, eliciting the patient's concerns and planning the encounter.^20^

According to the model,^20^ the first few moments of an encounter or meeting are key to establishing a trusting relationship. In our findings, there were examples of more or less constructive ways of starting family meetings. Small talk and addressing nonmedical issues to put the patient at ease seemed to be important. This corresponds with ‘creating a welcoming atmosphere’ in the FHM.^20^ Likewise, open‐ended questions, welcoming the patient's perspective and concerns, characterized the more successful meetings that we observed. We also found that explaining the purpose of the meeting to the patient and the other attendees before beginning the next stage appeared crucial, especially in creating predictability. According to Frankel and Stein,^20^ letting the patient know what to expect is a crucial part of ‘investing in the beginning’. Moreover, a central function of IC is to provide continuity in the patient pathway between the hospital and the home.^32^ In line with this, our results indicate that family meetings, when structured according to the FHM, may contribute to relational continuity, which aims to provide the patient with a sense of predictability and coherence (during their patient pathway).^5^, ^32^

According to Frankel and Stein,^20^ HCPs may overlook the importance of the first few moments of encounters with patients, creating an augmented sense of power imbalance between HCPs and patients and the risk of confusing the patient and hindering the chance to elicit the patient's perspective.^20^ For example, some of the observed meetings were started directly with the ‘what matters to you?’ question without first laying down the grounds for the family meeting. This could arguably serve to impede patient participation. In line with this, the theme ‘what matters to you?’ illustrated important features of the practice of patient participation as person‐centred goal setting in the IC family meetings. Here, eliciting the ‘full spectrum’ of concerns and seeing them in the context of the patient's total life situation appeared central. This is in line with the second habit in the FHM, ‘eliciting the patient's perspective’, which emphasizes assessing the patients' point of view on their problems and goals as well as the impact that their issues may have on their total life situation.^20^ One important pay‐off of this second habit^20^ is the uncovering of hidden concerns.

In line with previous research,^11^, ^33^ we found that HCPs gained new insights when applying the ‘what matters to you’ approach. Furthermore, our findings arguably showed that ‘digging deeper’ into what matters to the patient (i.e., not just eliciting what matters, but what matters *most*) is important. What the patient believes impedes or is necessary in meeting their goals seems important for real patient participation to occur from the family meetings. According to Frankel and Stein,^20^ several mistakes may be committed regarding eliciting the patient's perspective. For example, failure to elicit the full spectrum of concerns and their relative level of importance from the patient's viewpoint is not uncommon. The result may be that HCPs miss important concerns or that these concerns emerge very late in the meeting,^20^ thus hampering or delaying patient participation.

The third theme, ‘being empathetically present’, captured how HCPs managed to be empathetic with the patients to varying degrees and corresponded with the third habit in the FHM.^20^ This habit entails the sensitive assessment of the patient's body language and tone of voice as well as being aware of how one's own communication might facilitate the participation of the patient and make the encounter meaningful.^20^ Our findings show the varying degrees to which HCPs managed to be empathetically present with patients. There were examples of HCPs missing or overlooking patients' nonverbal cues or subtle verbal invitations to discuss concerns but also of clear empathetic communication. Different ways of communicating empathetically have been presented in previous research on the model.^20^, ^23^, ^31^ For example, we found that in some of the meetings, HCPs used both verbal and non‐verbal cues (head‐shaking, silence, ‘tell me more’, etc.), which can be referred to as ‘continuers’.^20^ These linguistic devices encourage the patient to elaborate on their perspective^20^ and may signal a genuine interest to the patients and their relatives. The *subtlety* by which especially older people express their concerns has been addressed in prior research.^34^, ^35^ A review by Murray et al.^34^ from a similar context showed how HCPs' misreading of older patients' expressions of their wish to participate inhibited their involvement in care.

Finally, we found that the final closure of the IC family meetings was particularly important. This entailed summing up the family meeting, checking the patient's comprehension and reaching agreement on the plan or future course of action, corresponding to the fourth habit in the FHM.^20^ The most important thing hampering final closure or ‘investment in the end’ was lack of attendance of people who were central to deciding on a future plan of action. In the current context, CMs were crucial to shared decision‐making, giving appropriate information and concluding the family meeting, which are all central parts of habit four.^20^ In addition, given the patients' age and the complexity of needs, relatives were also central but often missing from the meetings. This is supported by previous research on how relatives become central information brokers and representatives for the patient's voice for older people during their pathway and appear central to providing continuity of care.^36^, ^37^, ^38^

### 4.1 Practice implications

There seems to be potential for considerable improvements in the structuring of family meetings in IC and refining the communication skills used by HCPs in clinical practice. When family meetings follow the FHM, they have the potential to facilitate actual patient participation and the (three) different dimensions of continuity of care.^5^, ^6^ By establishing trust and exploring the patient's perspective and preferences, being sensitive to the patient's emotions and involving the patient in shared decision‐making, *relational continuity* ^5^, ^6^ may be achieved. Using the FHM in these meetings also has important implications for *informational continuity*.^5^, ^7^ In particular, ensuring that important information brokers (i.e., relatives who know the patient well) and decision‐makers (i.e., CMs) are present at the meetings seems crucial. Finally, by structuring family meetings in line with the FHM, HCPs in IC are also given a tool to facilitate *management continuity*,^5^, ^7^ which entails communication with HCPs across settings in the patient pathway from hospital to home. Overall, the FHM may help HCPs better structure family meetings and thus serve as a useful tool in daily practice.

### 4.2 Strengths and limitations

Using a theoretical framework in analysing the empirical data allowed us to go beyond the surface in an interpretative way, serving as a tool to bring coherence and depth to the study. However, when narrowing the lens using a specific framework such as the FHM, we might lose the possibility to see other dimensions of how patient participation was negotiated. The model focuses on communication at a microlevel. Although structural aspects and resources are important issues in the construction of patient participation, they are not discussed in this study. To our knowledge, we are the first to shed light on how patient participation is constructed within family meetings in IC services using the FHM as a theoretical framework. However, we cannot conclude on the effectiveness of the FHM. The validity of the research is strengthened using tape‐recorded nonparticipant observation, which gives a nuanced representation of what happened in the IC meetings.^39^, ^40^ In addition, the observed family meetings were completed by one researcher, who had a lot of clinical experience related to transitional care for older persons via IC. This prior experience served to facilitate access to and understanding of the field, but it also made the researcher ‘blind’ to certain aspects of the field.^40^, ^41^ The preunderstanding of the authors was that patient participation in IC is difficult to achieve and needs to be framed more competently. Therefore, to ensure reflexivity, the researchers engaged in reflexive discussions about the validity of the analysis and results with several other researcher/peers who were not as familiar with or engaged in the field.^40^, ^41^

Furthermore, to enhance validity, a memo was written to reflect on preconceptions and to keep track of the research process.^42^ A possible limitation may be that the author's attendance at the IC meetings could have affected the participants' behaviours (e.g., the HCPs may have boosted person‐centred communication to give a good impression of the IC practice).^42^, ^43^ The authors translated the quotes from the family meetings verbatim into English, controlled by a professional editor. However, since translation is an activity of both language and culture, there may be nuances in language that we have not captured. The low number of observations (*N* = 14) might be a limitation. However, after analysing all the meetings, the same patterns evolved across the meetings; thus, we assume sufficient information power. Regarding transferability, the issue is not whether the findings are generalizable to other settings in a positivistic sense, but rather the possible implications that the findings may have to other contexts.^41^, ^44^ Rich descriptions enhance transferability,^45^ and by providing such descriptions, we believe that our findings may be transferable beyond the immediate study context.^46^

## 5 CONCLUSION

This article has explored how patient participation is framed and negotiated within family meetings in IC through the lens of the FHM. We found considerable variation in the way current family meetings were conducted. The importance of investing in the beginning was illustrated by various ways of starting the meetings; it seemed crucial to start the meeting with a proper introduction and explanation of the purpose of the meeting to be able to successfully move to the next stage of eliciting the patients' preferences, views and goals via the ‘what matters to you?’ question. There were examples of empathetic communication among meeting participants, which was perceived to facilitate patient participation and care continuity. Finally, to successfully end the meeting and agree on a shared plan, it seemed crucial for CMs who held the decision‐making power to attend the meetings. Our results indicated that the most successful meetings to negotiate patient participation were the ones including all the four habits. Framing family meetings in line with the four habits sequential approach may have the potential to assure patient participation and care continuity in IC services. Future research should explore the effectiveness and feasibility of how teaching programmes incorporating the FHM into family meetings may improve communication skills to facilitate patient participation and continuity of care in IC.

## CONFLICT OF INTEREST

The authors declare no conflict of interest.

## Data Availability

As the data collection approval for the main study states the data to be available only to the researchers, data and materials collected for this manuscript will not be shared.

## References

[1] Pearson M , Hunt H , Cooper C , Sheppard S , Pawson R , Anderson R . Providing effective and preferred care closer to home: a realist review of intermediate care. Health Soc Care Community. 2015;23:577‐593. 10.1111/hsc.12183 25684035

[2] Martinsen B , Norlyk A , Lomborg K . Experiences of intermediate care among older people: a phenomenological study. Br J Community Nurs. 2015;20:74‐79. 10.12968/bjcn.2015.20.2.74 25651281

[3] Milte CM , Ratcliffe J , Davies O , Whitehead C , Masters S , Crotty M . Family meeting for older adults in intermediate care settings: the impact of patient cognitive impairment and other characteristics on shared decision making. Health Expect. 2015;18:1030‐1040. 10.1111/2Fhex.12076 23683120 PMC5060899

[4] Kvæl L , Debesay J , Bye A , Bergland A . The dramaturgical act of positioning within family meetings—negotiation of patients' participation in intermediate care services. Qual Health Res. 2020;30:811‐824. 10.1177/1049732319873054 31526100

[5] Haggerty JL , Reid RJ , Freeman GK , Starfield BH , Adair CE , McKendry R . Continuity of care: a multidisciplinary review. BMJ. 2003;327(7425):1219‐1221. 10.1136/bmj.327.7425.1219 14630762 PMC274066

[6] Gulliford M , Naithani S , Morgan M . What is ‘continuity of care’. J Health Serv Res Policy. 2006;11:248‐250. 10.1258/135581906778476490 17018200

[7] Health Quality Council of Alberta (HQCoA) . *Understanding patient and provider experiences with relationship. Information and management continuity*. Calgary, Alberta, Canada: Health Quality Council of Alberta; 2016. Accessed February 6, 2022. https://hqca.ca/studies-and-reviews/relationship-information-and-management-continuity/

[8] Griffith JC , Brosnan M , Lacey K , Keeling S , Wilkinson TJ . Familiy‐meetings—a qualitative exploration of improving care planning with older people and their families. Age Ageing. 2004;33:577‐581. 10.1093/ageing/afh198 15381505

[9] Kvæl L , Debesay J , Bye A , Langaas A , Bergland A . Choice, voice and co‐production in intermediate care: exploring geriatric patients' and their relatives' perspectives on patient participation. Sage Open. 2019;9:1‐13. 10.1177/2158244019876318 34290901

[10] Kvæl L , Debesay J , Bye A , Bergland A . Healthcare professionals' experiences of patient participation among older patients in intermediate care—at the intersection between profession, market and bureaucracy. Health Expect. 2019;22:921‐930. 10.1111/hex.12896 31127681 PMC6803410

[11] Olsen C , Debesay J , Bergland A , Bye A , Langaas A . What matters when asking, “what matters to you?”—perceptions and experiences of health care providers on involving older people in transitional care. BMC Health Serv Res. 2020;20:1‐13. 10.1186/s12913-020-05150-4 PMC7164237 32299424

[12] Lovdata . Lov om pasient‐ og brukerrettigheter [The Patients' Rights Act] LOV‐07‐02‐63). Oslo: helse‐ og omsorgsdepartementet;1999. Lov om pasient‐ og brukerrettigheter (pasient‐ og brukerrettighetsloven).

[13] Dent M , Pahor M . Patient involvement in Europe—a comparative framework. J Health Organ Manag. 2015;29:546‐555. 10.1108/jhom-05-2015-0078 26222875

[14] Kvæl L , Debesay J , Langaas A , Bye A , Bergland A . A concept analysis of patient participation in intermediate care. Patient Educ Couns. 2018;101:1337‐1350. 10.1016/j.pec.2018.03.005 29551564

[15] Davis S , Byers S , Walsh F . Measuring person‐centred care in a sub‐acute health care setting. Aust Health Rev. 2008;32:496‐504. 10.1071/AH080496 18666878

[16] Finset A . Emotions, narratives and empathy in clinical encounters. Int J Integr Care. 2010;10:53‐56. 10.5334/ijic.490 PMC2834911 20228917

[17] Papadimitriou C , Cott C . Client‐centred practices and work in inpatient rehabilitation teams: results from four case studies. Disabil Rehabil. 2015;37:1135‐1143. 10.3109/09638288.2014.955138 25163833

[18] Elbourne HF , le May A . Crafting intermediate care: one team's journey towards integration and innovation. J Res Nurs. 2015;20:56‐71. 10.1177/2F1744987114568656

[19] van Dongen JJJ , Habets IGJ , Beurskens A , von Bokhoven MA . Successful participation of patients in interprofessional team meetings: a qualitative study. Health Expect. 2017;20:724‐733. 10.1111/hex.12511 27714904 PMC5513000

[20] Frankel RM , Stein T . Getting the most out of the clinical encounter: the four habits model. Perm J. 1999;3:79‐88. 10.7812/TPP/99-020 11317576

[21] Jensen B , Gulbrandsen P , Dahl F , Krupta E , Frankel R , Finset A . Effectiveness of a short course in clinical communication skills for hospital doctors: results of a crossover randomized controlled trial. Patient Educ Couns. 2011;84:163‐169. 10.1016/j.pec.2010.08.028 21050695

[22] Mith R , Dwamena F , Frankel R . An evidence‐based patient‐centered method makes the biopsychosocial model scientific. Patient Educ Couns. 2013;91:265‐270. 10.1016/j.pec.2012.12.010 23352913

[23] Lundeby T , Gulbrandsen P , Finset A . The expanded four habits model—a teachable consultation model for encounters with patients in emotional distress. Patient Educ Couns. 2015;98:598‐603. 10.1016/j.pec.2015.01.015 25724347

[24] Castro EM , Regenmortel TV , Vanhaecht K , Semeus W , Hecke AV . Patient empowerment, patient participation and patient‐centeredness in hospital care: a concept analysis based on a literature review. Patient Educ Couns. 2016;99:1923‐1939. 10.1016/j.pec.2016.07.026 27450481

[25] Jull J , Köpke S , Smith M , et al. Decision coaching for people making healthcare decision. Cochrane Database Syst Rev. 2021;11:CD013385. 10.1002/14651858.cd013385.pub2 34749427 PMC8575556

[26] Ringard Å , Sagen A , Saunes IS , Lindahl AK . Norway: health system review. Health Syst Transit. 2013;15:1‐162. 24434287

[27] Rostgaard T . Quality reforms in Danish home care—balancing between standardisation and individualisation. Health Soc Care Community. 2012;20:247‐254. 10.1111/j.1365-2524.2012.01066.x 22512317

[28] Vabø M . Norwegian home care in transition—heading for accountability, off‐loading responsibilities. Health Soc Care Community. 2012;20:283‐291. 10.1111/j.1365-2524.2012.01058.x 22356449

[29] Braun V , Clarke V . Using thematic analysis in psychology. Qual Res Psychol. 2006;3:77‐101. 10.1191/1478088706qp063oa

[30] Runkle C , Wu E , Wang EC , Gordon GH , Frankel R . Clinician confidence about conversations at the end of life is strengthened using the four habits approach. J Psychosoc Oncol. 2008;26:81‐95. 10.1080/07347330802118040 19042266

[31] Krupat E , Frankel R , Stein T , Irish J . The Four Habits Coding Scheme: validation of an instrument to assess clinicians' communication behavior. Patient Educ Couns. 2016;62:38‐45. 10.1016/j.pec.2005.04.015 15964736

[32] Kvæl LAH , Hellesø R , Bergland A , Debesay J . Balancing standardisation and individualisation in transitional care pathways: a meta‐ethnography of the perspectives of older patients, informal caregivers and healthcare professionals. BMC Health Serv Res. 2022;22:430. 10.1186/s12913-022-07823-8 35365140 PMC8974038

[33] Nilsen ER , Hollister B , Söderhamn U , Dale B . What matters to older adults? Exploring person‐centred care during and after transitions between hospital and home. J Clin Nurs. 2022;31:569‐581. 10.1111/jocn.15914 34117673

[34] Murray J , Hardicre N , Birks Y , O'Hara J , Lawton R . How older people enact care involvement during transition from hospital to home: a systematic review and model. Health Expect. 2019;22:883‐893. 10.1111/hex.12930 31301114 PMC6803411

[35] Pols J . Enacting appreciations: beyond the patient perspective. Health Care Anal. 2005;13:203‐221. 10.1007/s10728-005-6448-6 16223211

[36] Aase K , Waring J . Crossing boundaries: establishing a framework for researching quality and safety in care transitions. Appl Ergon. 2020;89:103228. 10.1016/j.apergo.2020.103228 32763449

[37] Olsen CF , Bergland A , Bye A , Debesay J , Langaas AG . Crossing knowledge boundaries: health care providers' perceptions and experiences of what is important to achieve more person‐centered patient pathways for older people. BMC Health Serv Res. 2021;21:1‐16. 10.1186/s12913-021-06312-8 33827714 PMC8028726

[38] Dyrstad DN , Storm M . The role of next of kin in care transitions. In: Aase K , Waring J , Schibevaag L , eds. Researching Quality in Care Transitions. Springer; 2017:87‐101. 10.1007/978-3-319-62346-7_5

[39] Liu F , Maitlis S . Nonparticipant observation. In: Mills AJ , Durepos G , Wiebe E , eds. Encyclopedia of Case Study Research. Vol 2. SAGE; 2010.

[40] Moen K , Middelthon A . Qualitative research methods. In: Laake P , Benestad H , Olsen B , eds. Research in Medical and Biological Sciences—from Planning and Preparation to Grant Application and Publication. Elsevier; 2015.

[41] Malterud K . Qualitative research: standards, challenges, and guidelines. Lancet. 2001;358(9280):483‐488. 10.1016/s0140-6736(01)05627-6 11513933

[42] Maxwell JA . Qualitative Research Design: an Interactive Approach. 3rd ed. SAGE Publications; 2013:218.

[43] Morse JM . Critical analysis of strategies for determining rigor in qualitative inquiry. Qual Health Res. 2015;25:1212‐1222. 10.1177/1049732315588501 26184336

[44] Morse JM . Qualitative generalizability. Qual Health Res. 1999;9:5‐6. 10.1177/2F104973299129121622

[45] Lincoln YS , Guba EG . Naturalistic Inquiry. Sage Publications; 1985:416.

[46] Flyvbjerg B . Five misunderstandings about case‐study research. Qual Inq. 2006;12:219‐245. 10.1177/2F1077800405284363

